# Supplementary material for: Boosting potassium-ion batteries by few-layered composite anodes prepared via solution-triggered one-step shear exfoliation
Source: Nat Commun. 2018 Sep 7;9:3645. doi: 10.1038/s41467-018-05786-1 (PMC6128879; doi:10.1038/s41467-018-05786-1)
Supplement: Supplementary file 1 — Supplementary Information [file 41467_2018_5786_MOESM1_ESM.pdf]

# Supporting Information

## Boosting potassium ion battery performance by few-layered composite anode prepared via solution-triggered one-step shear exfoliation

Liu et al.

## Supplementary Figures

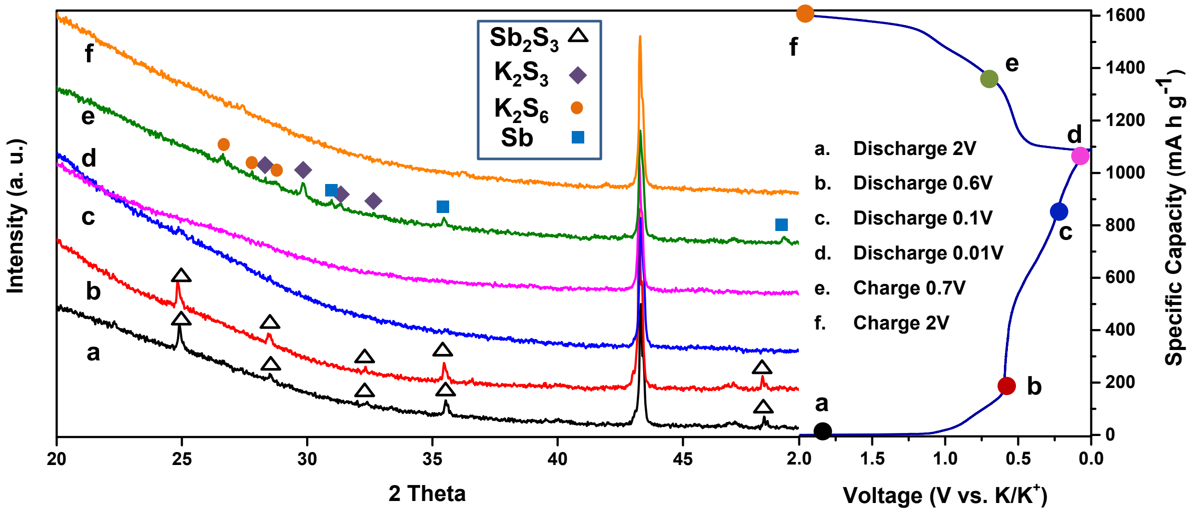


**Supplementary Fig. 1 Electrochemical mechanism of bulk $Sb_2S_3$**. Ex-situ XRD patterns of commercial bulk $Sb_2S_3$ at different potentials for the potassium ion battery

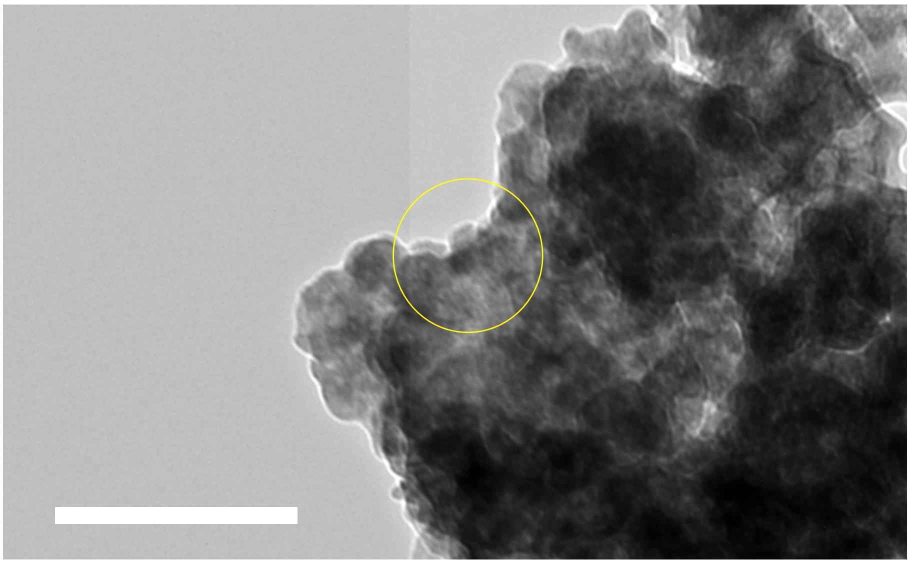

**Supplementary Fig. 2 Morphology of commercial $Sb_2S_3$ after discharge to 0.5 V.** TEM image of commercial $Sb_2S_3$ after discharge to 0.5 V with the yellow circle indicating the selected area for SAED in Fig. 1a. Scale bar: 100nm

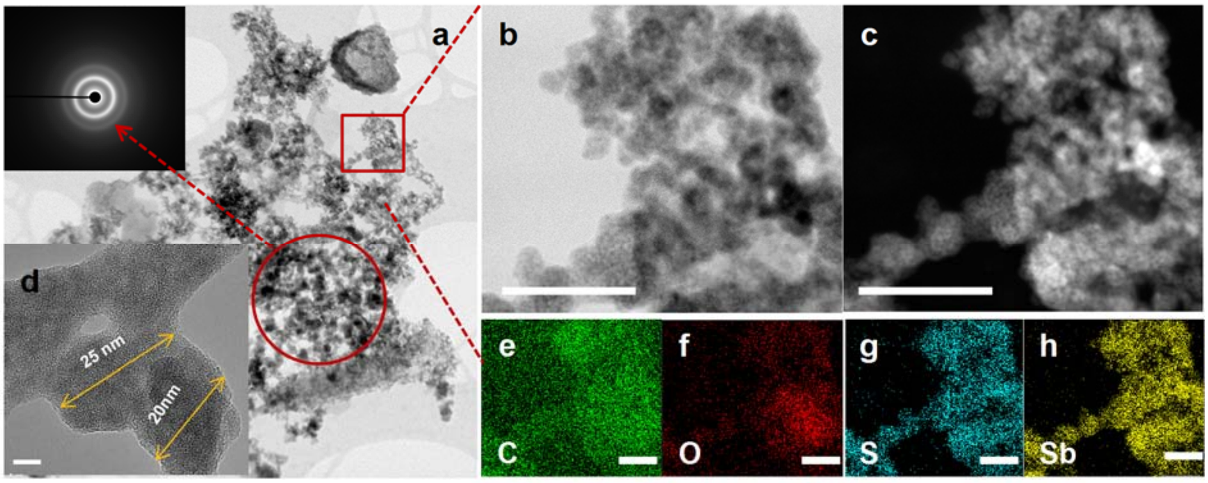


**Supplementary Fig. 3 Characterizations of bulk $Sb_2S_3$ after 1st charge by electron microscope.** TEM images and EDS mapping of bulk SBS after 1st charge. (a-c) Scanning TEM (STEM) images of the 1st charge product of commercial bulk $Sb_2S_3$ with inset SAED pattern of indicated area. (d) High-resolution TEM image of the bulk $Sb_2S_3$ after the 1st charge, and (e-h) corresponding EDX elemental mapping of carbon, oxygen, sulphur and antimony. Scale bars in b–h are 50, 50, 5, 50, 50, 50 and 50 nm, respectively.

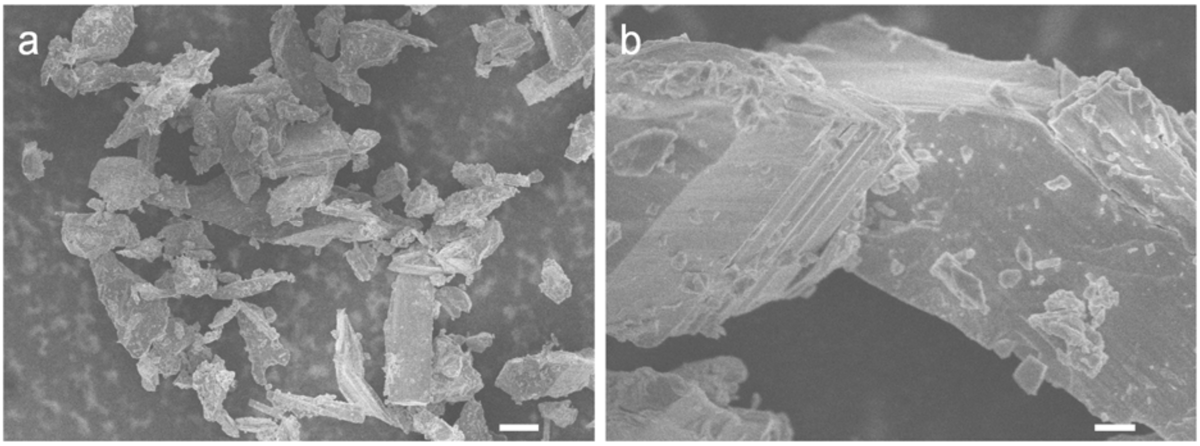


**Supplementary Fig. 4 Morphology of commercial bulk $Sb_2S_3$**. SEM images of commercial bulk $Sb_2S_3$. Scale bars: 10μm (a); 1μm (b)

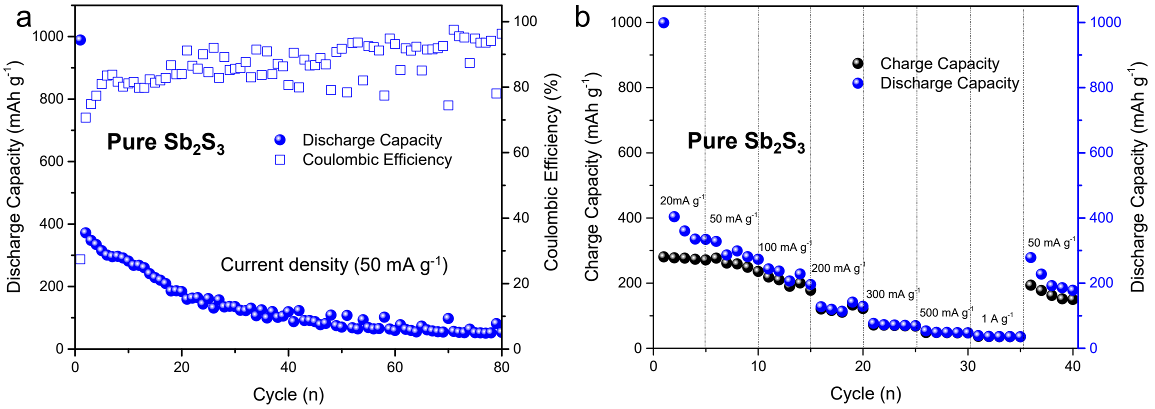


**Supplementary Fig. 5 Electrochemical performance of bulk SBS.** Cycling performance (a) and rate performance (b) of commercial bulk SBS.

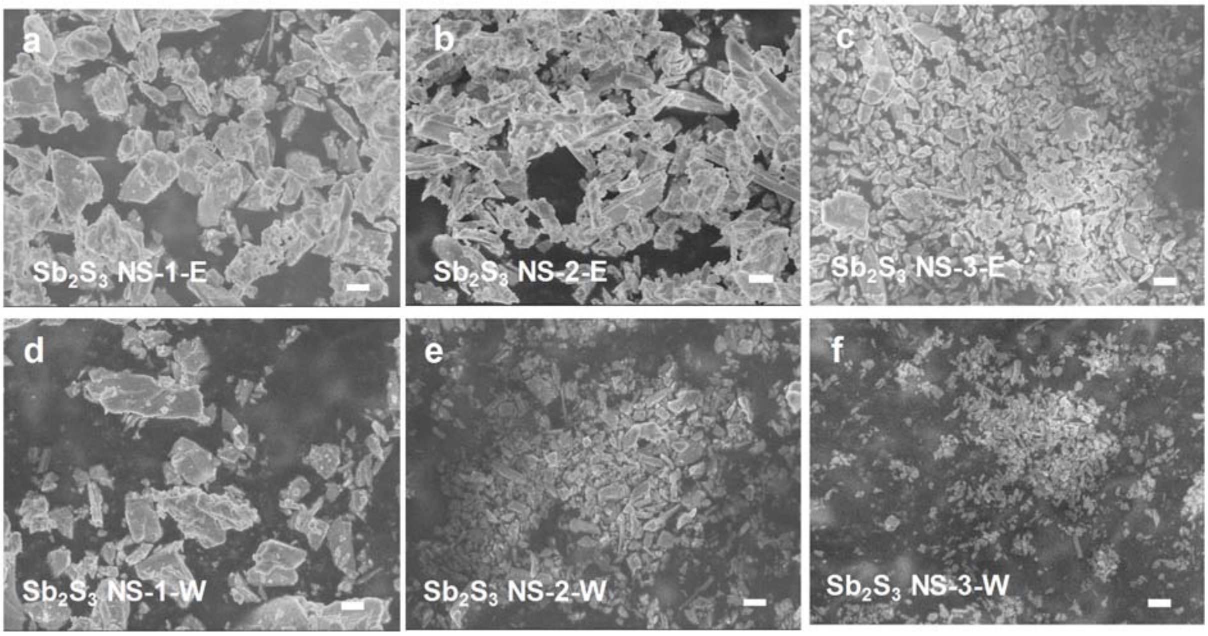


**Supplementary Fig. 6 SEM images of exfoliated SBS.** (a-c) SEM images of SBS exfoliated in ethanol from different sections of bottle (a: bottom; b: middle; c: top). (d-f) SEM images of SBS exfoliated in water in different sections (d: bottom; e: middle; f: top). Scale bars in (a-f) are all the same, 1μm.

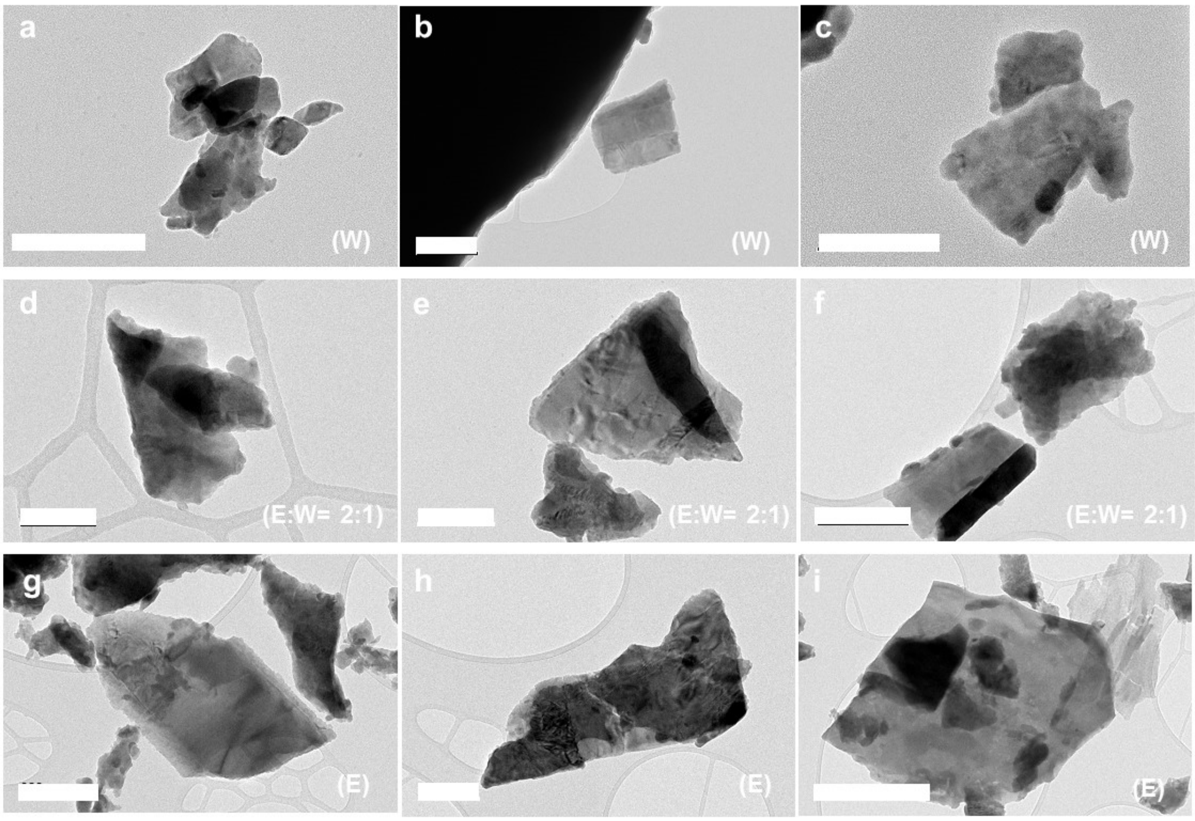


**Supplementary Fig. 7 Morphology of exfoliated SBS in different solution.** TEM images of layered SBS exfoliated from water (a-c), mixed solution of water and ethanol (d-f), and ethanol (g-i). Scale bars: 200 nm (a), 200 nm (b), 100nm (c), 100nm (d), 200 nm (e), 200 nm (f), 200 nm (g), 200 nm (h), 500 nm (i)

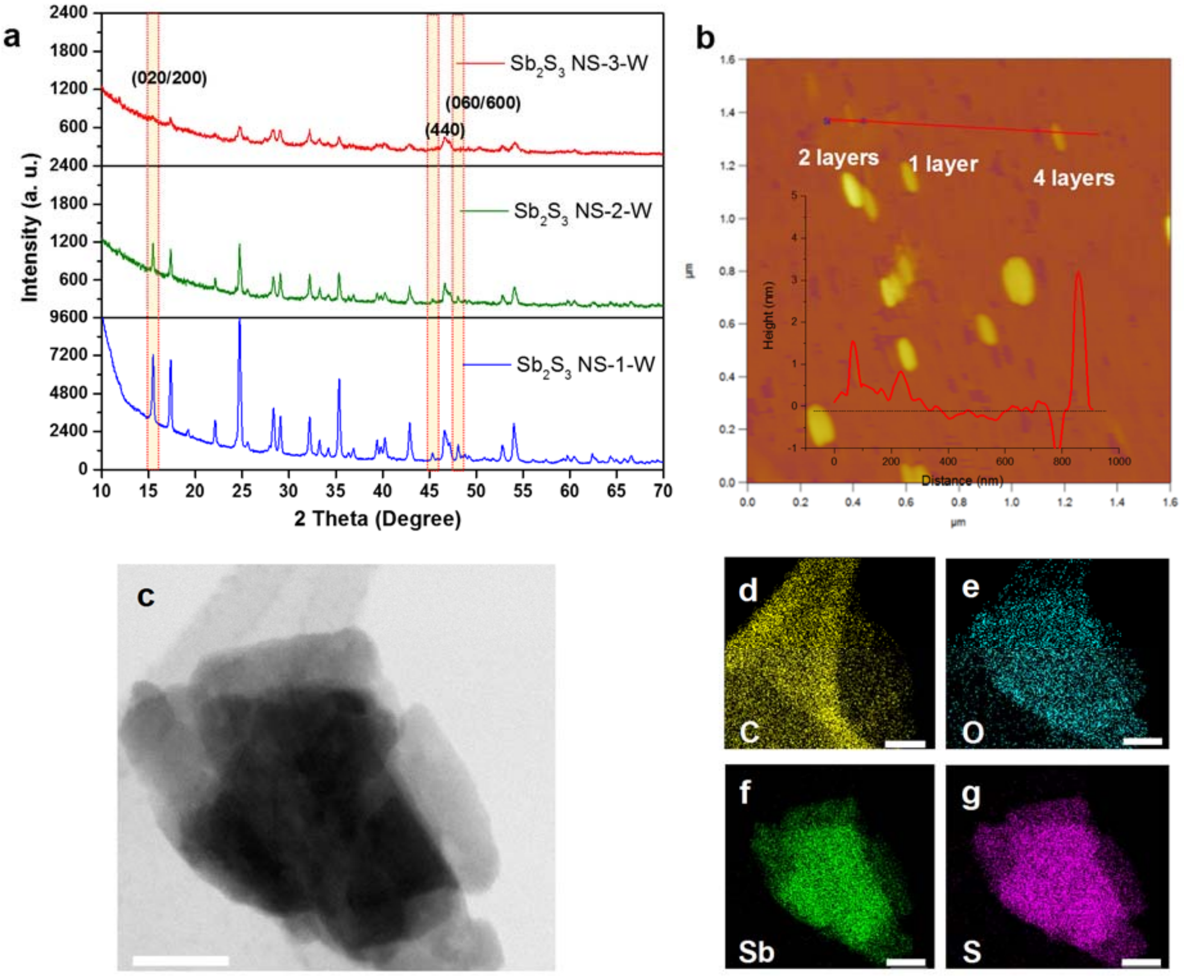


**Supplementary Fig. 8 Characterization of SBS exfoliated in water.** (a) XRD patterns of $Sb_2S_3$ in exfoliated in water and collected from different positions. (b) AFM images of $Sb_2S_3$ NS-3-W and cross-sectional height profiles with different layers of SBS. (c) TEM image of SBS nanosheets (NS-3-W) exfoliated in water. (d-g) Scanning TEM (STEM) images with EDX elemental mapping of sulphur, carbon, oxygen, and antimony. Scale bars: 50 nm (c); 50nm (d-g)

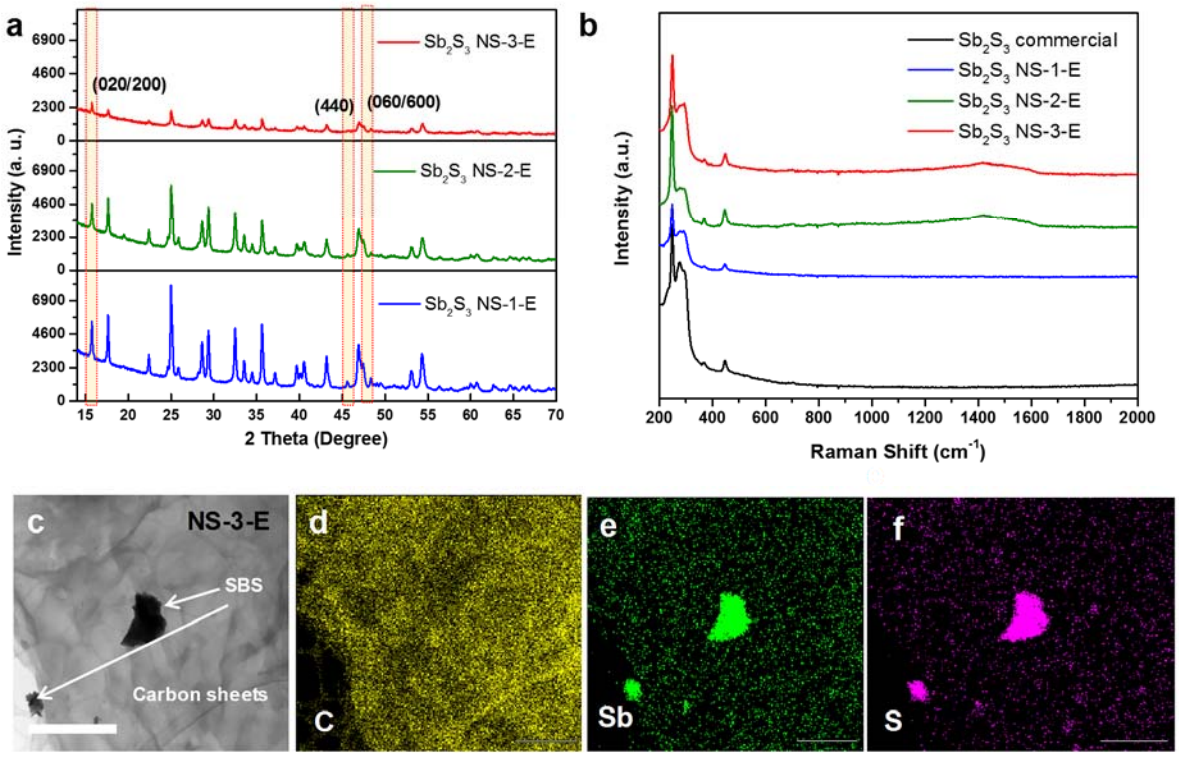


**Supplementary Fig. 9 Characterization of exfoliated SBS in ethanol.** (a) XRD patterns of $Sb_2S_3$ exfoliated in ethanol and collected from different positions. (b) Raman spectra of commercial SBS, SBS NS-1-E, SBS NS-2-E, and SBS NS-3-E. (c) TEM image of SBS/C composite and the indicated carbon sheets and SBS particles. (d-f) Scanning TEM (STEM) images with EDX elemental mapping of sulphur, carbon, and antimony. Scale bars: 2 μm (c-f)

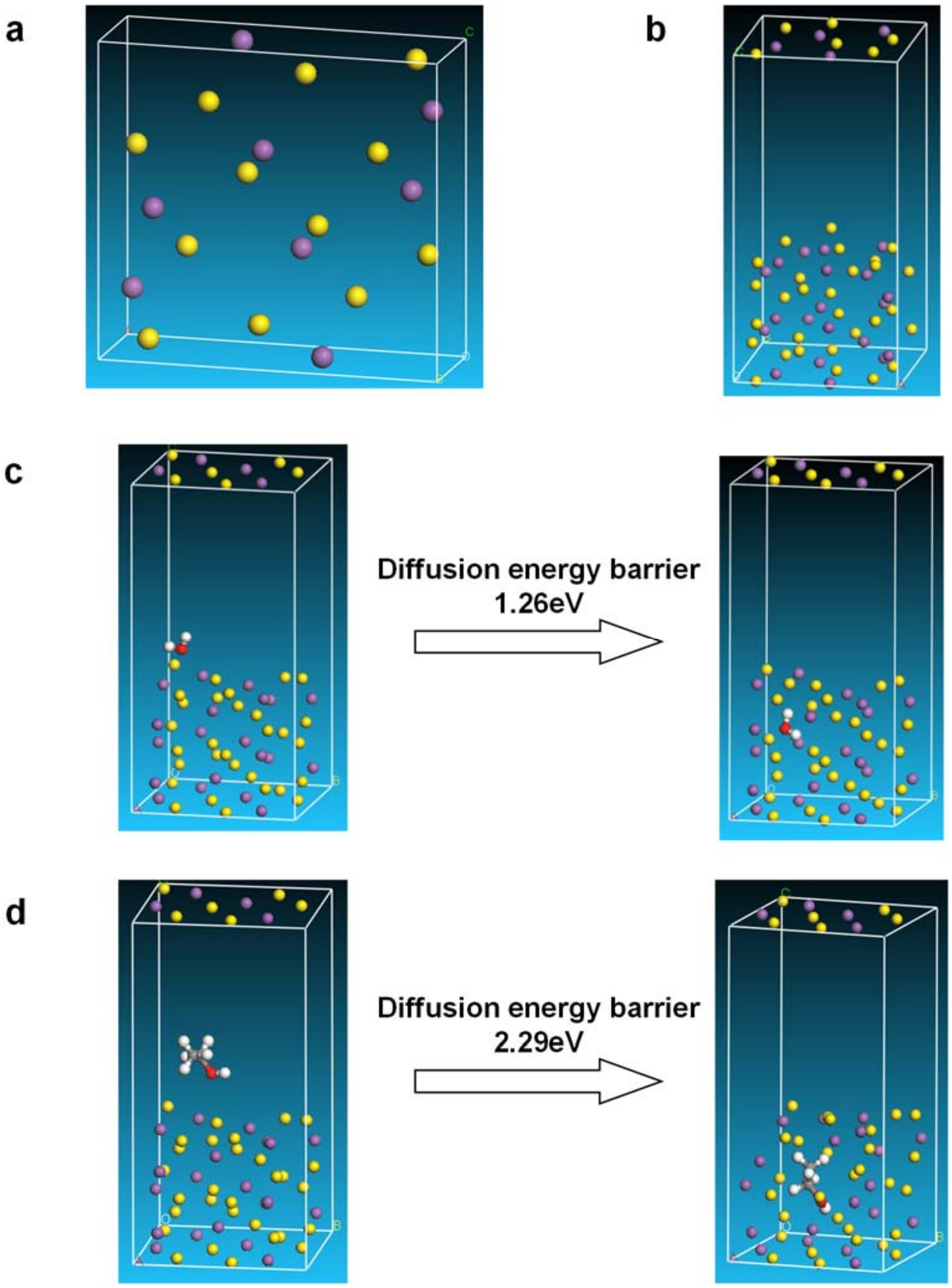


**Supplementary Fig. 10 DFT calculation of diffusion energy barrier of water and ethanol in SBS.** (a) $Sb_2S_3$ crystal cell model. (b) $Sb_2S_3$ (010) surface supercell model with five atomic layer thickness and 20 Å vacuum space. The relaxed initial and final states of $H_2O$ (c) and $CH_3CH_2OH$ (d) diffusion from the surface of SBS (010) into the position between the second and the third layer of an $Sb_2S_3$ (110) slab along the [010] direction.

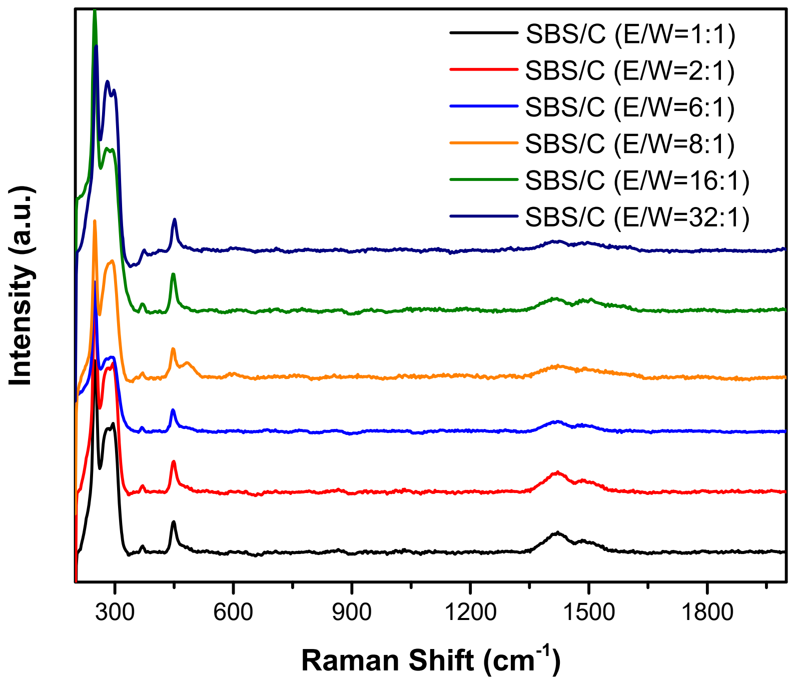


**Supplementary Fig. 11 Raman test of as-prepared SBS/C composites via one-step shear exfoliation.** Raman spectrum of SBS/C composite exfoliated in different solvents.

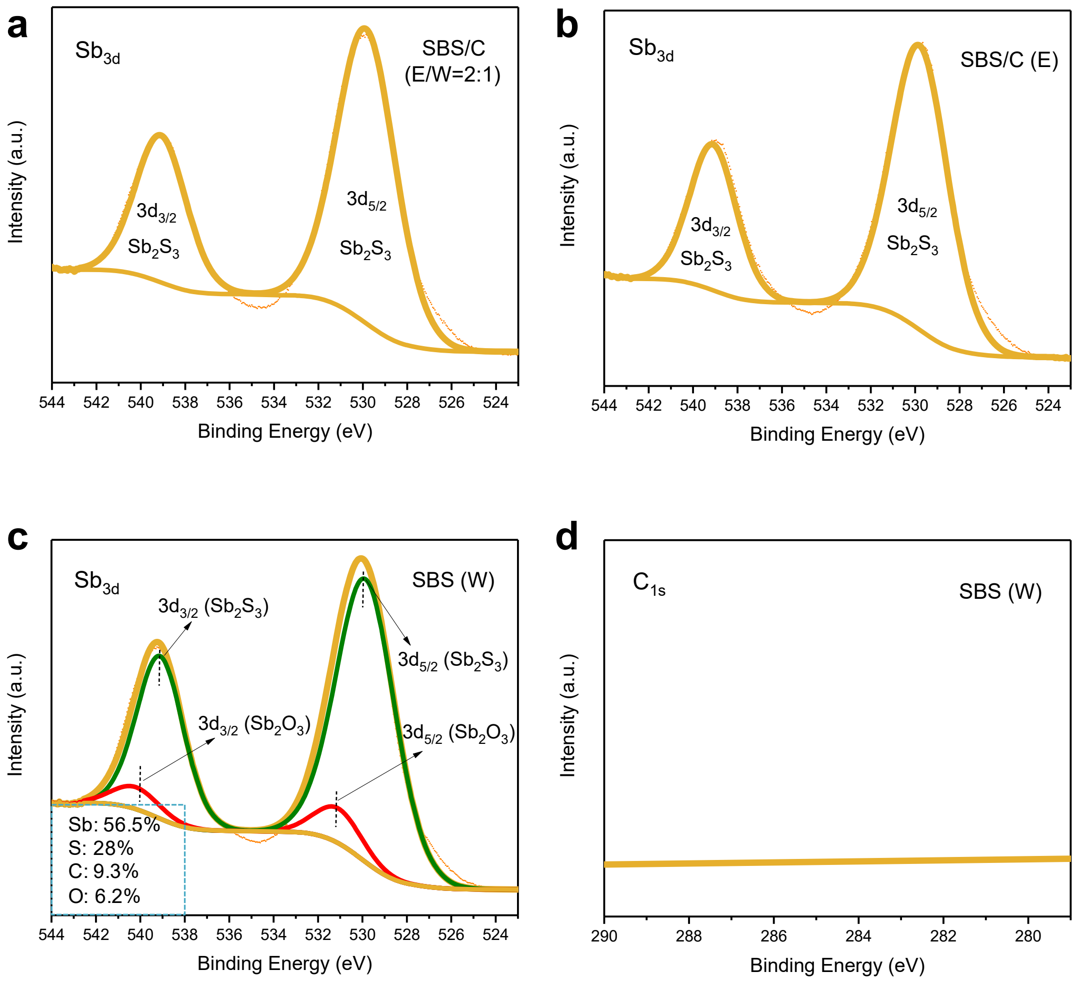


**Supplementary Fig. 12 Surface chemistry of SBS/C (E/W=2:1), SBS/C (E) and SBS (W).** XPS analysis of Sb 3d and C 1s peaks. Sb 3d peaks of (a) SBS/C (E/W = 2:1), (b) SBS/C (E), and (c) SBS/C (W); XPS analysis of C 1s peaks of (d) SBS/C (W).

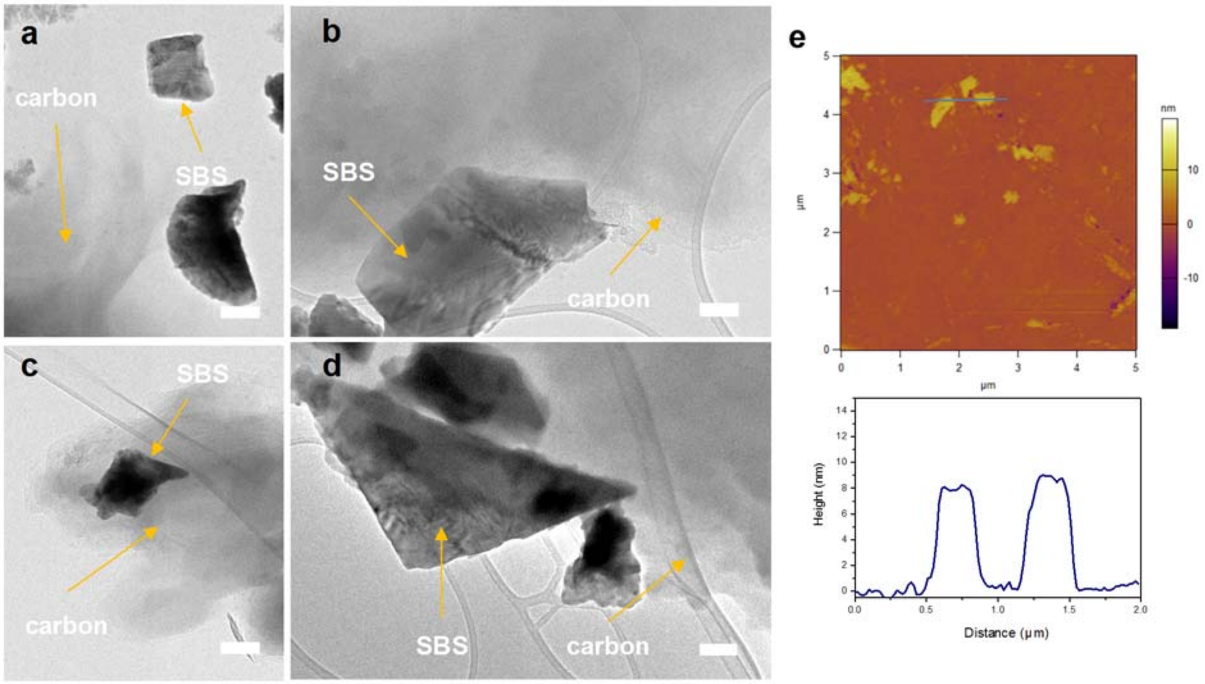


**Supplementary Fig. 13 Morphology of as-prepared layered SBS/C (E/W=2:1) composite.** (a-d) TEM images of SBS/C composite. (e) Typical AFM images with cross-sectional height profiles of SBS nanosheets. Scale bars: 200 nm (a); 100 nm (b); 200 nm (c); 100 nm (d)

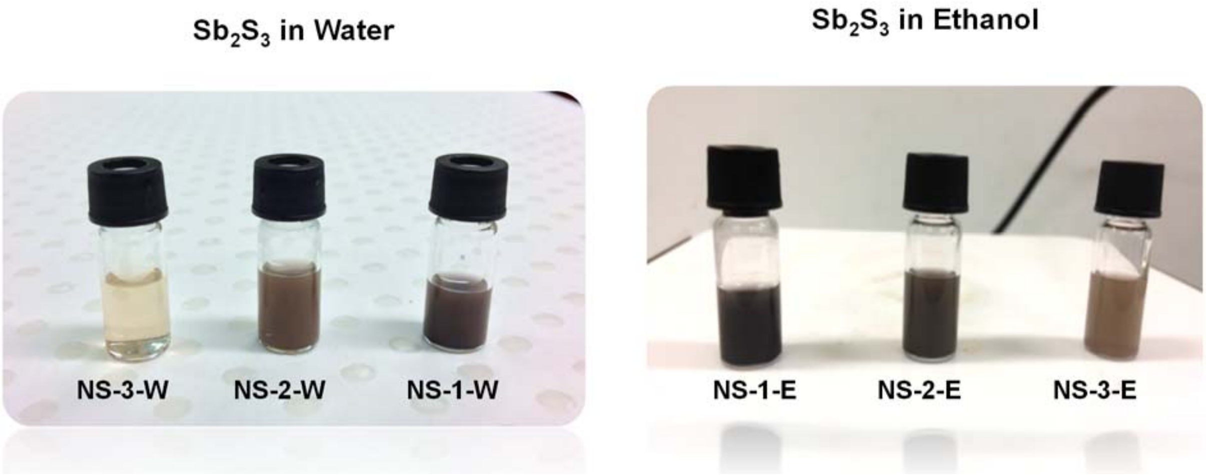


**Supplementary Fig. 14 Visual inspection of the exfoliated SBS.** Color variation due to the different size and thickness of $Sb_2S_3$ for samples collected at different positions for one solution, water on the left and ethanol on the right.

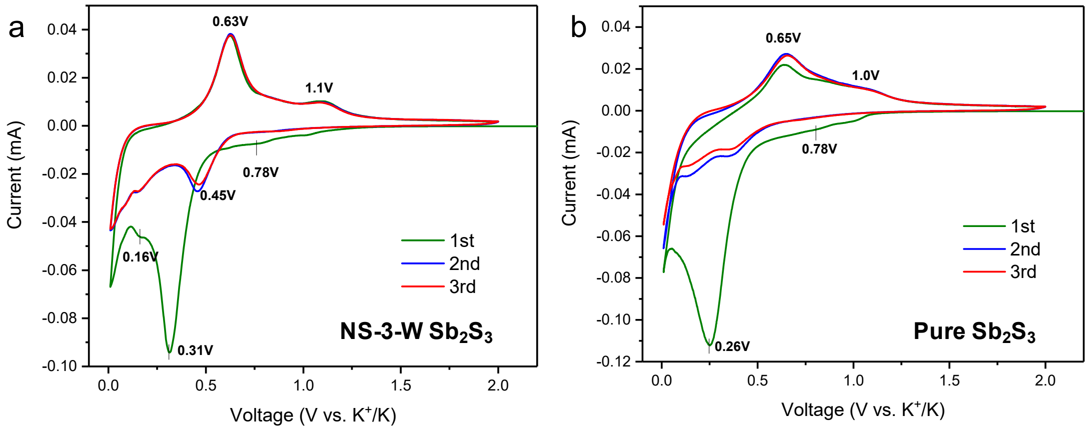


**Supplementary Fig. 15 Comparison of CV curves between SBS NS-3-W and bulk SBS.** CV curves of SBS NS-3-W (a) and commercial bulk SBS (b).

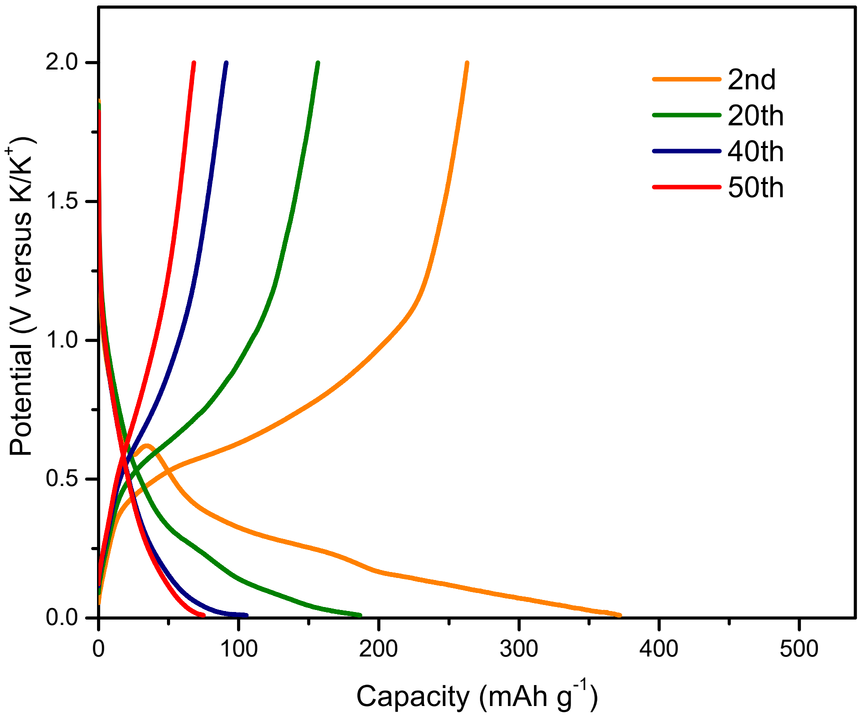


**Supplementary Fig. 16 Galvanostatic Discharge/charge profile of bulk SBS.** Discharge and charge curves for selected cycles of bulk SBS at 50 mA $g^{-1}$.

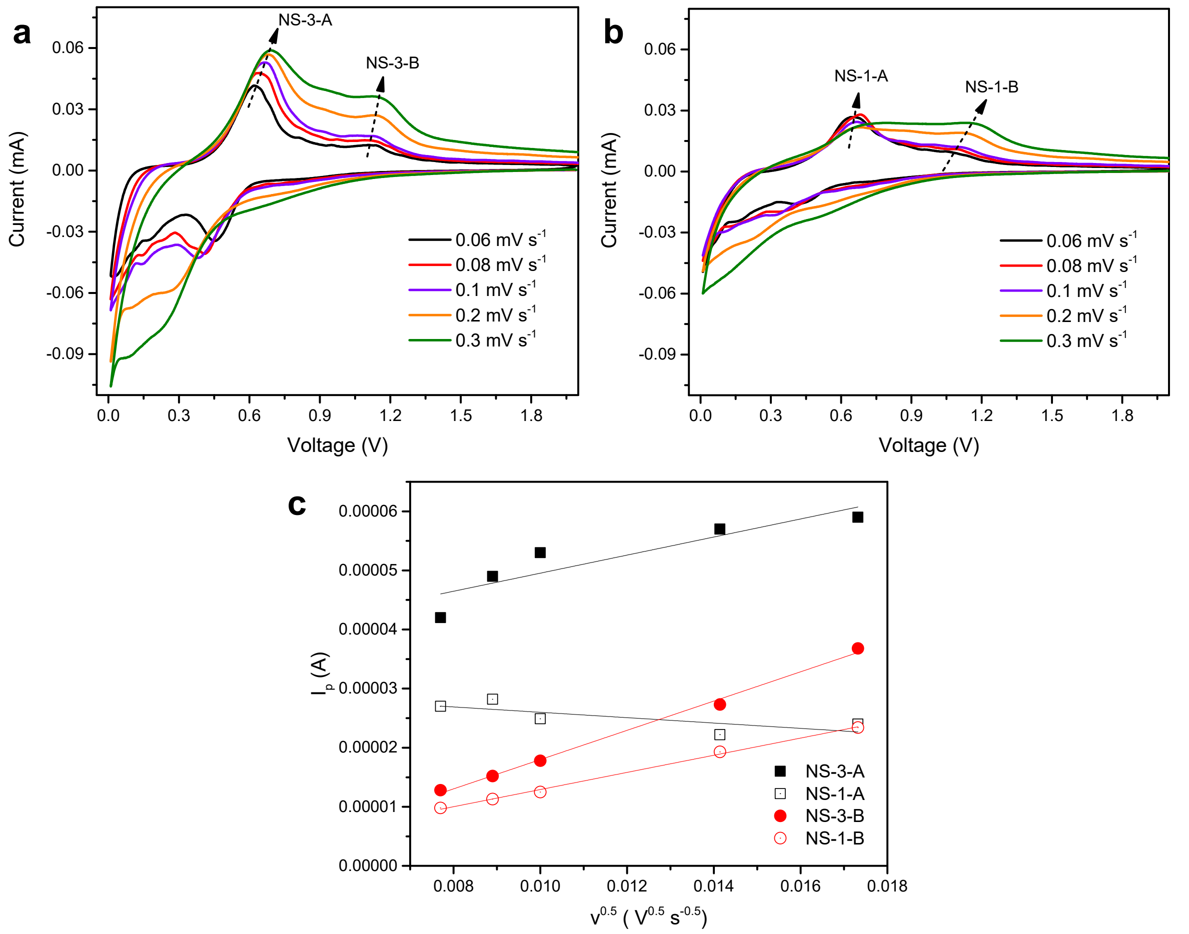


**Supplementary Fig. 17 The comparison of potassium-ion diffusion coefficient.** CV curves at different scan rates of SBS electrode: (a) NS-3-W and (b) NW-1-W. (c) Peak currents versus $V^{0.5}$ $s^{-0.5}$ and the corresponding linear fits.

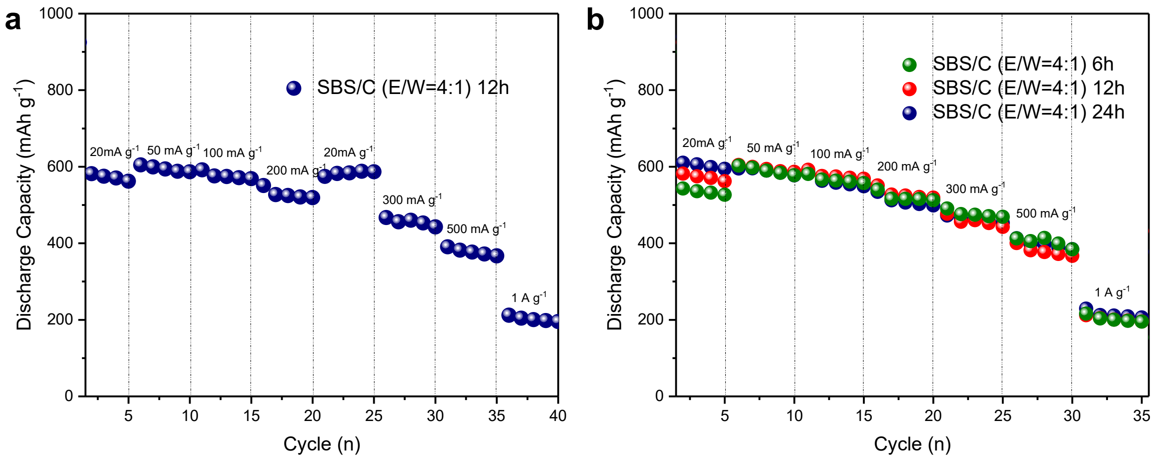


**Supplementary Fig. 18 Rate test under certain settings and with different standing times before testing**. (a) Rate performance of SBS/C (E/W = 4:1) with standing time of 12 h before the test (at the current densities of **20**, 50, 100, 200, **20**, 300, 500 mA $g^{-1}$, and 1 A $g^{-1}$). (b) Rate performance of SBS/C (E/W = 4:1) electrodes with different standing times (6 h, 12 h, 24 h).

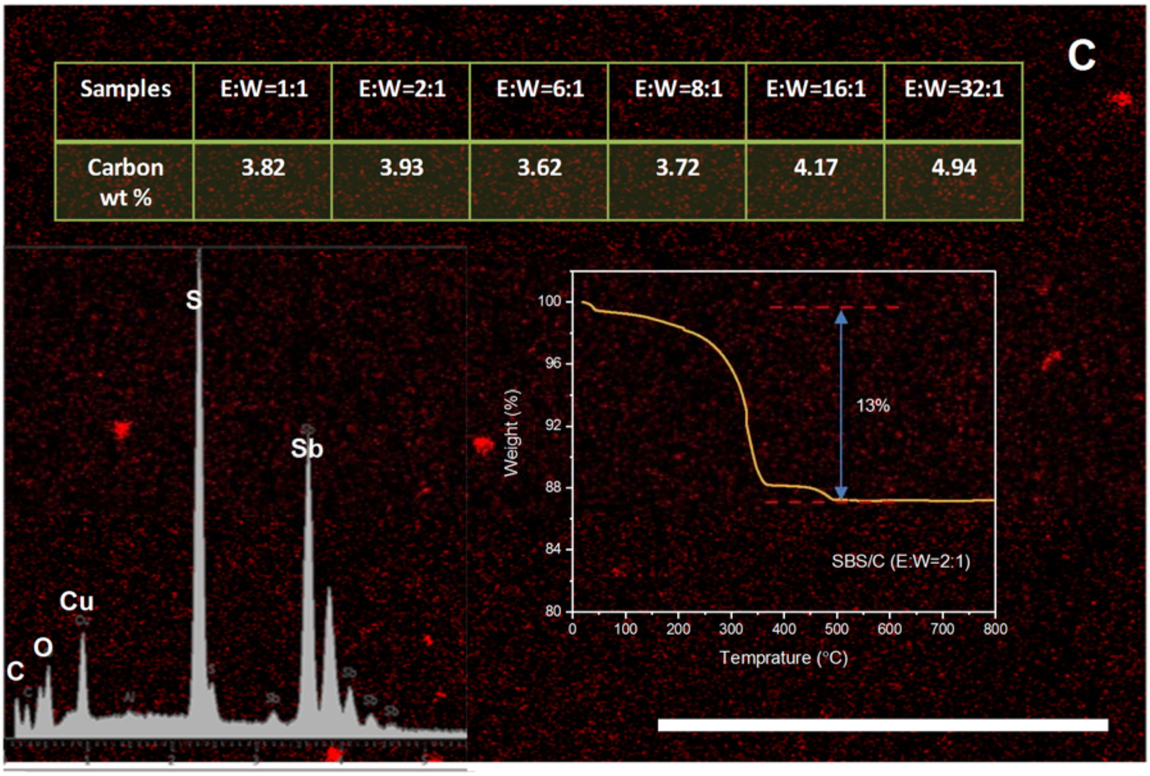


| Samples | E:W=1:1 | E:W=2:1 | E:W=6:1 | E:W=8:1 | E:W=16:1 | E:W=32:1 |
|---|---|---|---|---|---|---|
| Carbon wt % | 3.82 | 3.93 | 3.62 | 3.72 | 4.17 | 4.94 |

**Supplementary Fig. 19 Carbon determination in various SBS/C electrodes.** EDS mapping and inset spectrum showing the presence of carbon (E/W = 2:1); the inset table shows the carbon weight percentage for the composites of SBS/C (E/W = 1:1), SBS/C (E/W = 2:1), SBS/C (E/W = 6:1), SBS/C (E/W = 8:1), SBS/C (E/W = 16:1) and SBS/C (E/W = 32:1). Scale bar: 100 μm

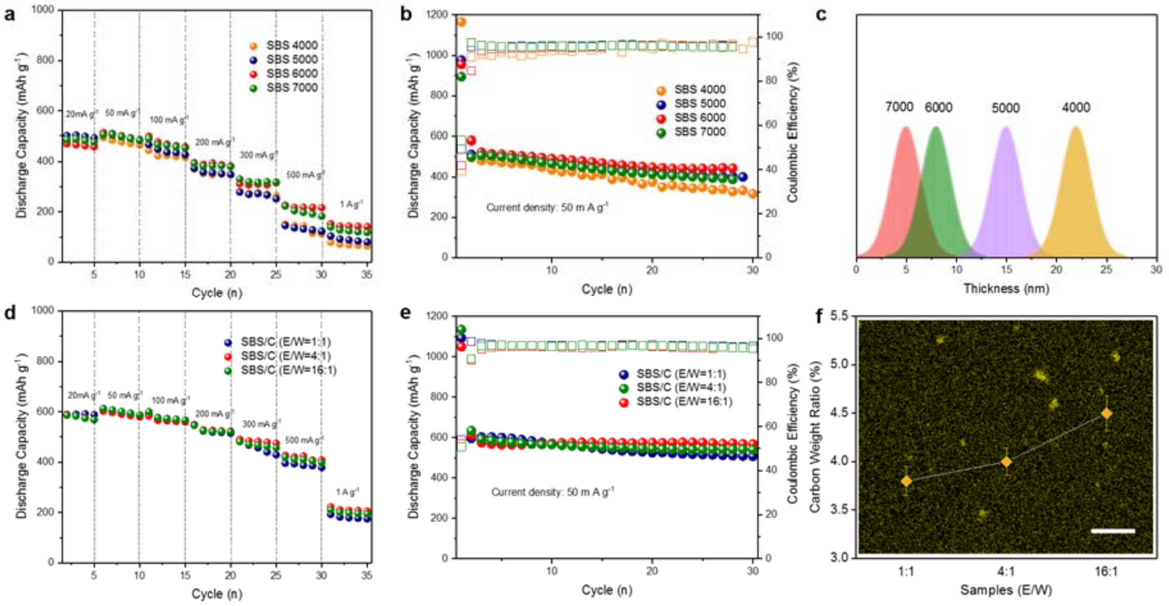


**Supplementary Fig. 20 Electrochemical properties of few-layered SBS and SBS/C electrodes fabricated via two-step exfoliation**. Comparison of (a) cycling performance and (b) rate performance of SBS 4000, SBS 5000, SBS 6000, and SBS 7000 electrodes, which were exfoliated in water with different rotation rates. (c) Thickness distributions for SBS 4000, SBS 5000, SBS 6000, and SBS 7000 electrodes. Comparison of (d) cycling performance and (e) rate performance of SBS/C (E/W = 1:1), SBS/C (E/W = 4:1), and SBS/C (E/W = 16:1) electrodes, which were exfoliated via two-step exfoliation (based on the exfoliated SBS 6000 products, with different weight ratios of ethanol added for the second step exfoliation). (e) Carbon weight percentages of the SBS/C (E/W = 1:1), SBS/C (E/W = 4:1), and SBS/C (E/W = 16:1) samples. Scale bar: 25 μm (f)

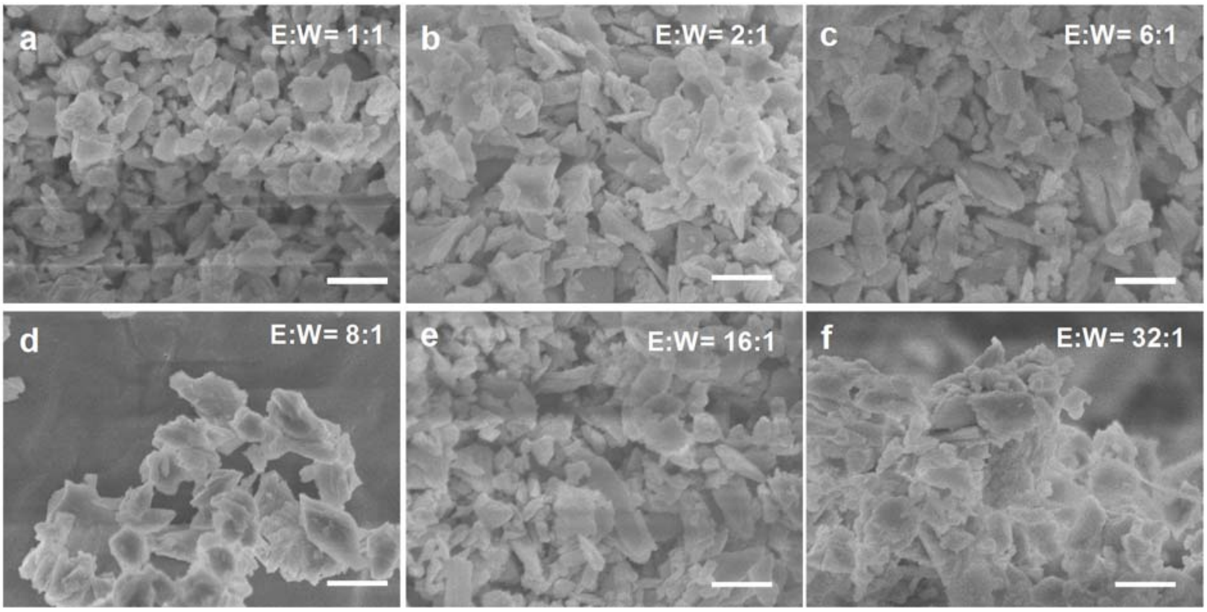


**Supplementary Fig. 21 Morphology of exfoliated SBS/C composites.** SEM images of exfoliated SBS with the different solvents (E/W = 1:1, E/W = 2:1, E/W = 6:1, E/W = 8:1, E/W = 16:1, E/W = 32:1). Scale bars: 1 μm (a-f)

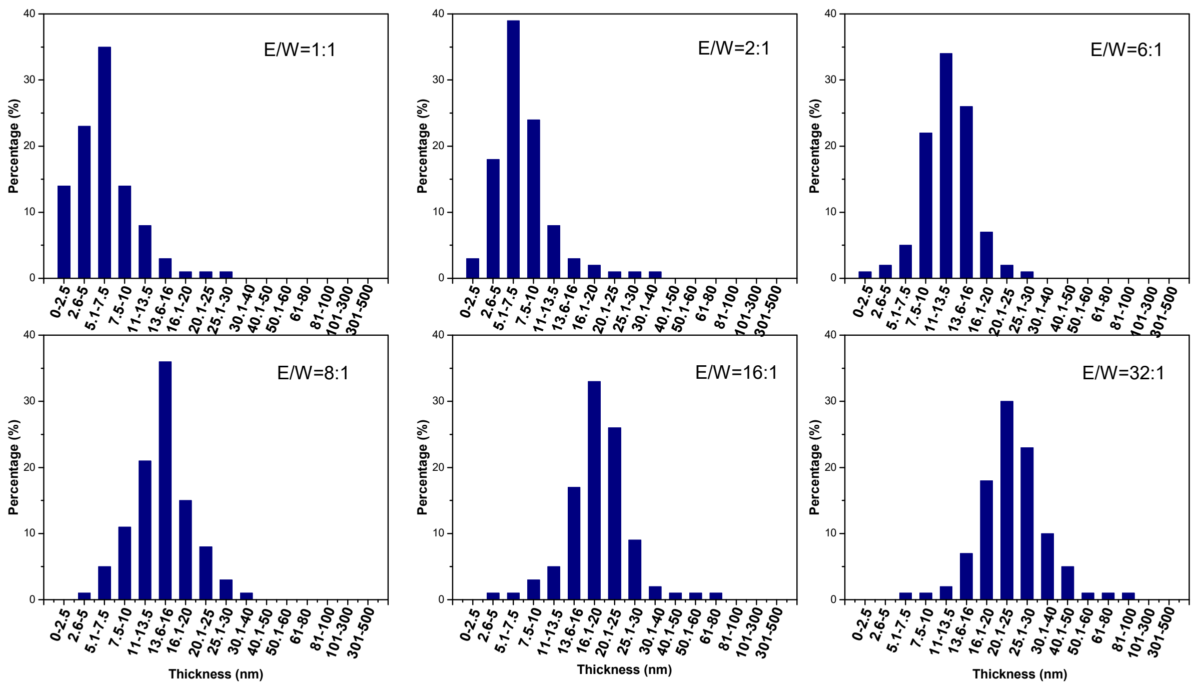


**Supplementary Fig. 22 The statistical thickness distributions of as-prepared SBS electrodes.** The exfoliated products in different exfoliation solvents with different ratios of ethanol to water.

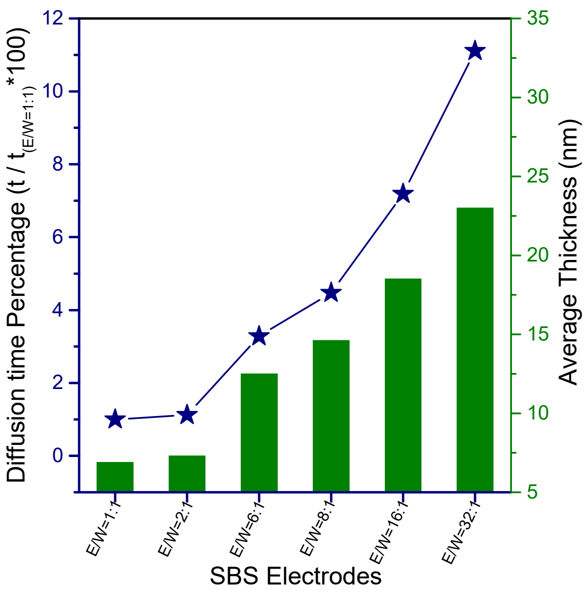


**Supplementary Fig. 23 Comparison of K ion diffusion times for SBS/C electrodes.** K ion diffusion time and the average thickness of different layered SBS electrodes (SBS/C (E/W = 1:1), SBS/C (E/W = 2:1), SBS/C (E/W = 6:1), SBS/C (E/W = 8:1), SBS/C (E/W = 16:1), SBS/C (E/W = 32:1)).

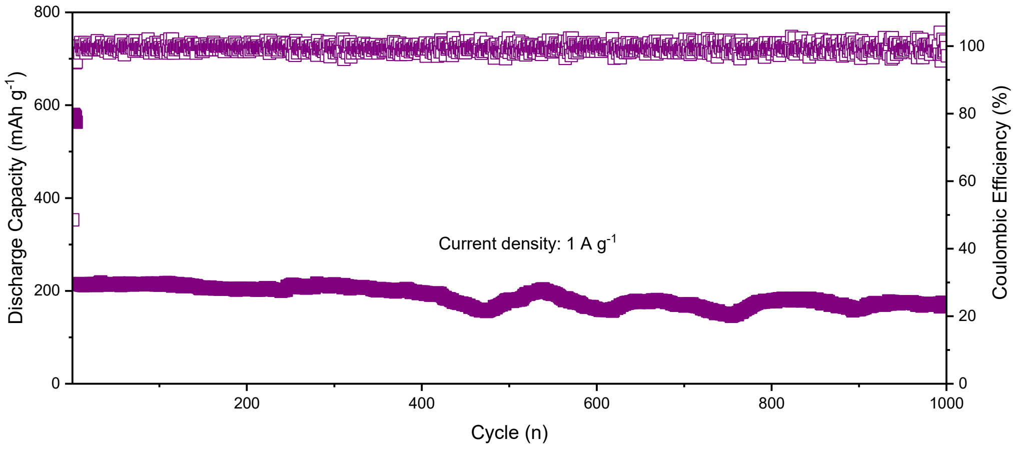


**Supplementary Fig. 24 Cycling stability of SBS/C (E/W=2:1) electrode.** Long-term cycling performance of SBS/C (E/W = 2:1) electrode at the current density of 1A $g^{-1}$

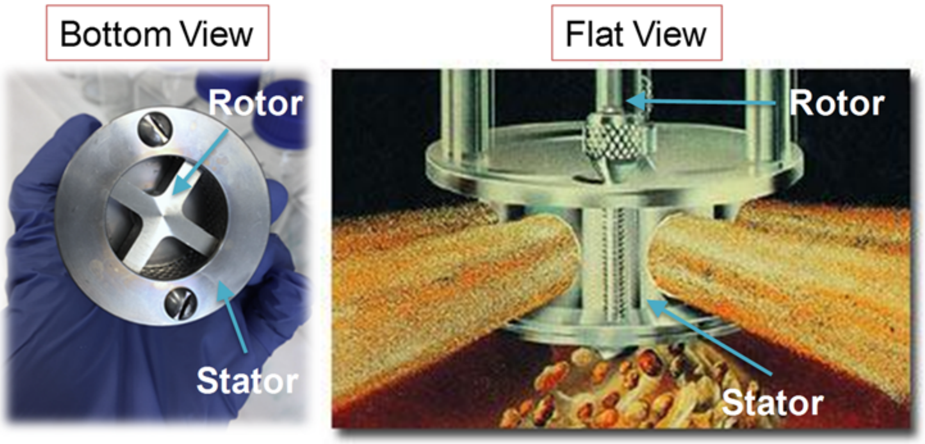


**Supplementary Fig. 25 Mixing head of exfoliation machine.** Bottom view (left) and side view (right) of mixing head with indicated rotor and stator.

# Supplementary Tables

**Supplementary Table 1. XPS analysis of electrode surface after 50th charge**

| Samples | Content after fitting | | | | |
|---|---|---|---|---|---|
| | S (total) | S ($-SO_2-$) | S ($S^{2-}$) | S ($S^0$) | S ($S^0$)/S ($S^{2-}$) |
| SBS/C (E) | 1 | 33% | 57.76% | 9.24% | 16.5% |
| SBS (bulk) | 1 | 45% | 50.44% | 4.56% | 9.04 % |

# Supplementary Notes

**Supplementary Note 1 |** In order to understand the difference between water and ethanol in the ability to exfoliate $Sb_2S_3$, an $Sb_2S_3$ (010) surface model with a thickness of five atomic layers were firstly constructed based on the relaxed $Sb_2S_3$ crystal cell model (Supplementary Fig. 10a-b). The bottom two layers of atoms were fixed, and the vacuum space for the G surface was set at 20 Å to avoid coupling between the adjacent layers. Then, the adsorption and diffusion behaviour of single water or ethanol molecules on the $Sb_2S_3$ (010) surface were investigated.

The adsorption energies of a single water or ethanol molecule on the $Sb_2S_3$ (010) surface were calculated by Supplementary Equations (1) and (2), respectively.

$$E_{ads}\,[Sb_2S_3\ (010) + H_2O] = E_{tot}\,[Sb_2S_3\ (010) + H_2O] - E_{tot}\,[Sb_2S_3(010)] - E_{tot}\,(H_2O) \quad (1)$$

$$E_{ads}\,[Sb_2S_3\ (010) + C_2H_6O] = E_{tot}\,[Sb_2S_3\ (010) + C_2H_6O] - E_{tot}\,[Sb_2S_3(010)] - E_{tot}\,(C_2H_6O) \quad (2)$$

where $E_{tot}$ [$Sb_2S_3$(010) + $H_2O$] and $E_{tot}$ [$Sb_2S_3$(010) + $C_2H_6O$] refer to the total energies of the $Sb_2S_3$(010) surface with single water or ethanol molecule adsorption, respectively. $E_{tot}$ [$Sb_2S_3$ (010)] refers to the total energy of clear $Sb_2S_3$ (010) surface. $E_{tot}$ ($H_2O$) and $E_{tot}$ ($C_2H_6O$) refer to the total energy of a single water or ethanol molecule, respectively. The calculated results (presented in Fig. 2e) indicate that water tends to be more easily adsorbed on the (010) surface of $Sb_2S_3$ than ethanol.

Then, the diffusion energy barriers of water and ethanol molecules were further calculated by adopting the linear synchronous transit/quadratic synchronous transit (LST/QST) tools. The relaxed adsorption models of a single water or ethanol molecule on the $Sb_2S_3$ (010) surface (see the left-hand models of Supplementary Fig. 10c-d) were set as the initial state of the diffusion reaction, and the relaxed insertion models of a single water or ethanol molecule moving into the position between the second and the third layers of $Sb_2S_3$ (010) slabs (see the right-hand models of Figure S8c-d) were set as the final state of the diffusion reaction. The calculated diffusion energy barrier for insertion of a water molecule into $Sb_2S_3$ (010) slabs (1.2 6eV) is smaller than that of ethanol (2.2 9eV), suggesting that the path along the [010] direction is more accessible for water diffusion than ethanol diffusion.

**Supplementary Note 2 |** The quantitative sulfur analysis by XPS (Supplementary Table 1) was performed using the relative sensitivity factor method[1]. The atomic concentration ratio of $S^0$ to $S^{2-}$ could be obtained according to Equation (1), as shown below:

$$n_{S^0}/n_{S^{2-}} = (I_{S^0}/S_{S^0}) / (I_{S^{2-}}/S_{S^{2-}}) \quad (1)$$

where n is the number of atoms per $cm^3$, $I$ is the peak area, and $S$ the sensitivity factor.

After fitting the S 2p peaks for the SBS/C and SBS electrodes, each state of sulphur content could be estimated and the ratio of elemental $S^0$ to $S^{2-}$ on the electrode surface after the $50^{th}$ charge could be calculated as 16.5% and 9.04% for SBS/C (E) and SBS (bulk), respectively.

**Supplementary Note 3 |** From supplementary Fig. 17, the potassium-ion diffusion coefficient ($D_{K+}$) can be calculated based on the Randles-Sevcik equation:

$$i_p = 0.4463nF\sqrt{nFD/RT}AC\sqrt{v}$$

Where $i_p$ represents peak current, $n$ is the number of electrons, $F$ is the Faraday constant, $R$ is the gas constant, $T$ is the temperature, $A$ is the surface area of the electrode, $D$ is the diffusion coefficient, $C$ stands for the concentration of potassium-ions in the electrolyte, and $v$ is the voltage scanning rate.

**Supplementary Note 4 |** (In order to give reasonable and accurate results for the carbon content, we pressed the composite samples on copper film, and collected more than 10 points in low magnification in order to get the average values of carbon content.); the inset TG curves of the composite (SBS/C (E/W = 2:1)) in air with a heating rate of 4 °C $min^{-1}$ demonstrate the reliable results in EDS (considering that $Sb_2S_3$ is converted into $Sb_2O_4$ when heated to 800 °C in air, the carbon content in the composite (SBS/C (E:W=2:1)) is calculated to be about 4.5 wt%.

# Supplementary References:

1 Moulder, J. F., Sticle, W. F., Sobol, P. E. & Bomben, K. D. *Handbook of X-ray Photoelectron Spectroscopy.* (Perkin-Elmer Co., Eden Prairie, MN, 1992).
